# Propylene Glycol Alginate Sodium Sulfate Alleviates Cerulein-Induced Acute Pancreatitis by Modulating the MEK/ERK Pathway in Mice

**DOI:** 10.3390/md15020045

**Published:** 2017-02-17

**Authors:** Hui Zhang, Yueyue Li, Linqiang Li, Hua Liu, Liangkai Hu, Ying Dai, Jianqing Chen, Shuqi Xu, Weimin Chen, Xiaorong Xu, Xuanfu Xu

**Affiliations:** 1 Department of Gastroenterology, the Tenth People’s Hospital of Shanghai, Tongji University, Shanghai 200072, China; viannezhzxz@163.com (H.Z.); yueyueliYYL@163.com (Y.L.); llqleeoe@163.com (L.L.); liuhuamd@163.com (H.L.); 2 Department of Gastroenterology, the Shidong Hospital of Shanghai, Shanghai 200072, China; huliangkai800116@163.com (L.H.); dynchengmo@163.com (Y.D.); cjqing1202@163.com (J.C.); leqi@163.com (S.X.); tutu1118@sohu.com (W.C.)

**Keywords:** acute pancreatitis, apoptosis, autophagy, MEK/ERK pathway, PSS

## Abstract

Previous studies have focused on the effects of propylene glycol alginate sodium sulfate (PSS) against thrombosis, but the anti-inflammatory potential is unknown. Therefore, we specifically focused on the protective effects of PSS on cerulein-induced acute pancreatitis (AP) using a mouse model, and investigated the mechanism of PSS on autophagy and apoptosis via the Mitogen-activated protein kinase (MEK)/extracellular signal-regulated kinase (ERK) pathway. Cerulein (100 ug/kg) was used to induce AP by ten intraperitoneal injections at hourly intervals in Balb/C mice. Pretreatment with vehicle or PSS was carried out 1 h before the first cerulein injection and two doses (25 mg/kg and 50 mg/kg) of PSS were injected intraperitoneally. The severity of AP was assessed by pathological score, biochemistry, pro-inflammatory cytokine levels, myeloperoxidase (MPO) activity and MEK/ERK activity. Furthermore, pancreatic histological scores, serum amylase and lipase activities, tumor necrosis factor-α (TNF-α), interleukin (IL)-1β interleukin (IL)-6 levels, and MPO activity were significantly reduced by PSS via up-regulated MEK/ERK activity. The representative molecules of apoptosis and autophagy, such as Bcl-2, Bax, Lc-3, Beclin-1, P62, were remarkably reduced. Taken together, these results indicate that PSS attenuates pancreas injury by inhibiting autophagy and apoptosis through a mechanism involving the MEK/ERK signaling pathway.

## 1. Introduction

Acute pancreatitis (AP) is an acute inflammation that begins with acinar activation and culminates in varying degrees of severity including multiple organ failure and death caused by consecutive extra-acinar events. The development of AP is a multistep process. The acinar activation, which occurs in pancreatic acinar cells, includes the activation of zymogens and the release of pro-inflammatory cytokines such as tumor necrosis factor-α (TNF-α), interleukin (IL)-1β, and IL-6. TNF-a plays a pivotal role in the initiation and spread of inflammatory cascade to other organs and subsequent systemic complications. Besides exacerbating pancreatic acinar cell injury, TNF-a can also induce infiltration of neutrophils and initiate or aggravate the cascade of other proinflammatory cytokines. So it can be said, when AP occurs, TNF-α can initiate the inflammatory response [1]. The main mechanism involves the induction of a large number of inflammatory factors, such as IL-1 and IL-6, which can damage endothelial cells, increase local microcirculatory disorders, vascular permeability, and further aggravate pancreatic injury. The extra-acinar events include the pancreatic inflammatory response. In summary, AP is launched in two steps in general: first, intracellular enzyme activation occurs resulting in acinar cell injury. Then, a pancreatic inflammatory response occurs [2].

Propylene glycol alginate sodium sulfate (PSS), prepared by the chemical sulfation of low-molecular-weight alginate, one of the most abundant marine polysaccharides isolated from brown algae, has been commonly used as an anti-cardiovascular disease drug in China for nearly 30 years [3]. In recent years, with the development of clinical medication and the identification of the efficacy of PSS, research has gradually increased, especially regarding the molecular mechanism of PSS. This study is to verify the anti-inflammatory properties of PSS in acute pancreatitis.

Mitogen-activated protein kinase (MEK)/extracellular signal-regulated kinase (ERK) plays a fundamental role in the inflammatory response, and many different types of drugs play a vital role in modulating the MEK/ERK pathway. Previous studies have confirmed that the pathogenesis of AP caused by cerulein involved apoptosis and autophagy [4,5,6,7]. Therefore, we are to present that PSS exerts its pharmacological activities through the MEK/ERK pathway in relation to apoptosis and autophagy.

In the present study, we investigated the effects of PSS on cerulein-induced AP in a mouse model and further examined the anti-apoptosis and anti-autophagy mechanism of PSS. For a better insight into the protective effects, we investigated the effect of PSS on pancreatic digestive enzyme levels, histological changes, myeloperoxidase (MPO) activity, and the levels of pro-inflammatory mediators including TNF-α, IL-1β and IL-6.

## 2. Results

### 2.1. PSS Does Not Affect Pancreatic Function

**Figure 1:**
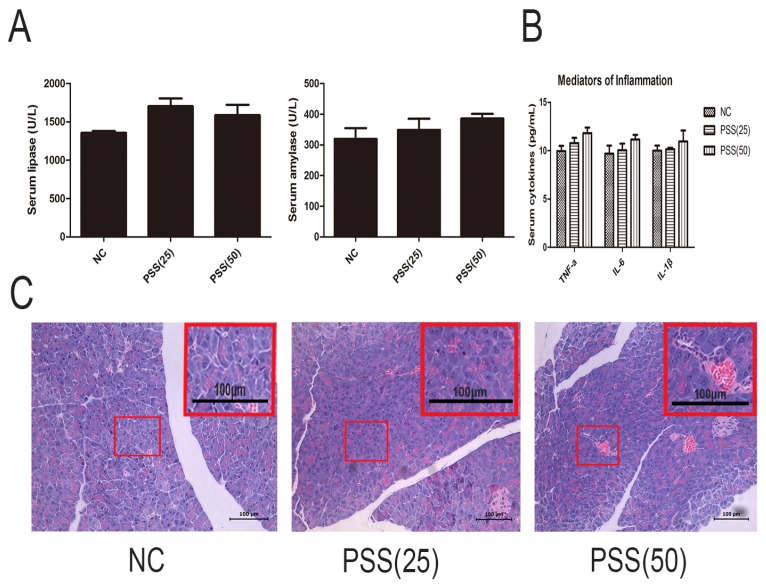
Effects of propylene glycol alginate sodium sulfate (PSS) on acute pancreatitis (AP)-induced enzyme production and inflammatory response. (**A**) The levels of serum amylase and lipase in the three groups did not differ. Data are expressed as means ± SD (*n* = 5, *p* > 0.05); (**B**) The serum levels of TNF-α, IL-6, and IL-1β in the three groups (*n* = 5, *p* > 0.05); (**C**) Hematoxylin and eosin-stained sections of the pancreas. Original magnification, ×200. A field of view was selected to magnify the observation of pancreatic tissue structure.

PSS itself may affect pancreatic function; therefore, we analyzed the effects of PSS on cytokine release and enzymes. Figure 1A shows that the levels of serum amylase and lipase did not differ between the three groups (*p* > 0.05), and the levels of TNF-α, IL-6, and IL-1βwere consistent (*p* > 0.05) (Figure 1B). Hematoxylin and eosin (H&E) staining, showed no obvious pathological change in any of the slices (Figure 1C).

### 2.2. PSS Pretreatment Attenuates Cerulein-Induced Acute Pancreatitis

**Figure 2:**
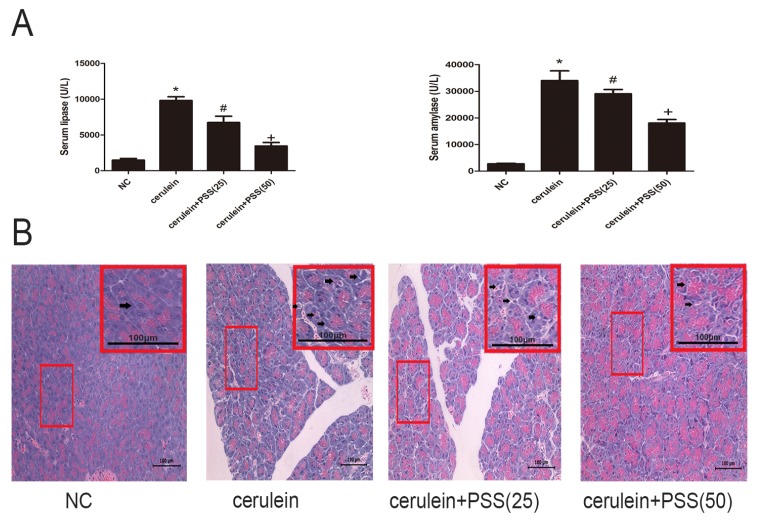
Effects of PSS on AP-induced enzyme production and pathology. (**A**) The levels of serum amylase and lipase changed depending on the PSS dose (25 mg/kg or 50 mg/kg). (*n* = 5, * *p* < 0.05 for Normal control (NC) versus cerulein, ^#^ *p* < 0.05 for cerulein + PSS (25 mg/kg) versus cerulein, and ^+^ *p* < 0.05 for cerulein + PSS (50 mg/kg) versus cerulein). (**B**) Representative hematoxylin and eosin-stained sections of the pancreas. The representative acinar edema, vacuolization, inflammatory cells infiltration and acinar cell necrosis were indicated with arrows.

Serum amylase and lipase are commonly used biochemical markers in AP. Pretreatment with PSS reduced the serum levels of amylase and lipase (*p* < 0.05), and the higher dose was more effective (Figure 2A). Figure 2B shows similar results in the histopathological study. Focal acinar cell degeneration, inflammatory cell infiltration, and necrosis were decreased in the PSS group compared with the AP group based on the result of Figure 2B. The histopathological score of the AP group was markedly higher than the NC group (*p* < 0.05) and the PSS treated group was observably lower than the AP group (*p* < 0.05) (Table 1). The changes were more marked in the high dose group. In sum, these results indicated that pretreatment with PSS effectively reduced cerulein-induced AP in mice.

### 2.3. PSS Pretreatment Inhibits the Production of TNF-α, IL-6, and IL-1β in Cerulein-Induced Acute Pancreatitis

**Figure 3:**
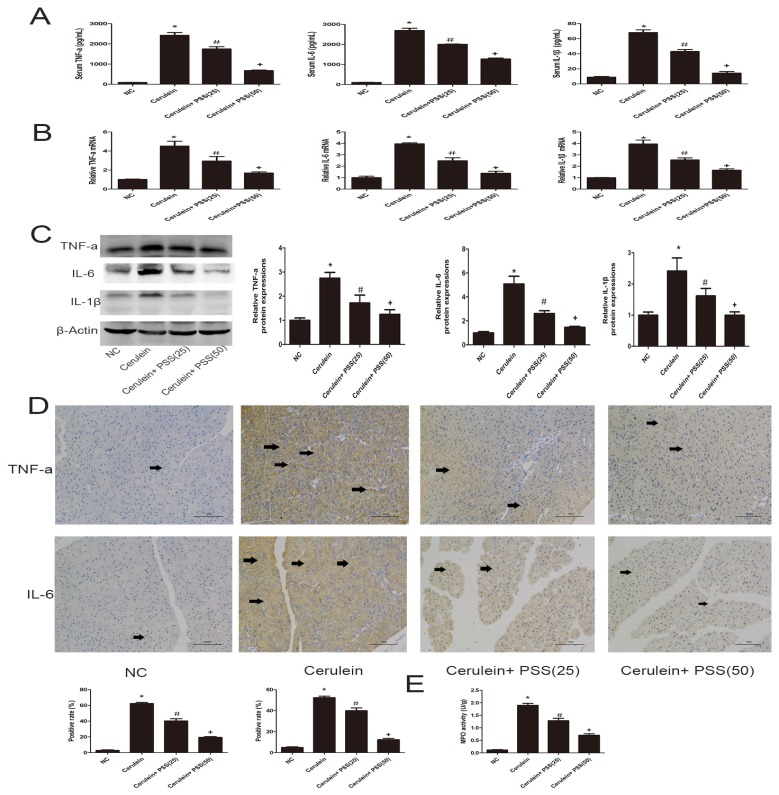
Effects of PSS on the production of TNF-α, IL-6 and IL-1β. (**A**) The levels of serum TNF-α, IL-6, and IL-1β measured by ELISAs were reduced by PSS pretreatment in mice at the doses of 25 mg/kg and 50 mg/kg. Data are expressed as means ± SD (*n* = 5, * *p* < 0.05 for NC versus cerulein, ^#^ *p* < 0.05 for cerulein + PSS (25 mg/kg) versus cerulein, and ^+^ *p* < 0.05 for cerulein + PSS (50 mg/kg) versus cerulein); (**B**) The mRNA levels of TNF-α, IL-6, and IL-1β were evaluated in each group by real-time PCR (*n* = 5, * *p* < 0.05 for NC versus cerulein, ^#^ *p* < 0.05 for cerulein + PSS (25 mg/kg) versus cerulein, and ^+^ *p* < 0.05 for cerulein + PSS (50 mg/kg) versus cerulein); (**C**) The expression levels of TNF-α, IL-6, and IL-1β proteins were determined by Western blotting and the gray values were calculated (*n* = 5, * *p* < 0.05 for NC versus cerulein, ^#^ *p* < 0.05 for cerulein + PSS (25 mg/kg) versus cerulein, and ^+^ *p* < 0.05 for cerulein + PSS (50 mg/kg) versus cerulein); (**D**) Immunohistochemistry staining (×200) showing the expression of TNF-α and IL-6. The ratio of brown area to total area was analyzed (*n* = 5, * *p* < 0.05 for NC versus cerulein, ^#^ *p* < 0.05 for cerulein + PSS (25 mg/kg) versus cerulein, and ^+^ *p* < 0.05 for cerulein + PSS (50 mg/kg) versus cerulein). The representative positive cells were indicated with arrows; (**E**) The myeloperoxidase activity, measured by a myeloperoxidase (MPO) assay kit, was reduced by PSS pretreatment in mice at the doses of 25 mg/kg and 50 mg/kg. Data are expressed as means ± SD (*n* = 5, * *p* < 0.05 for NC versus cerulein, ^#^ *p* < 0.05 for cerulein + PSS (25 mg/kg) versus cerulein, and ^+^ *p* < 0.05 for cerulein + PSS (50 mg/kg) versus cerulein).

The release of pro-inflammatory cytokines, including TNF-α, IL-6, and IL-1β, is closely associated with the development of AP. The levels of TNF-α, IL-6, and IL-1β were determined by ELISA and found to be clearly increased in the AP group (*p* < 0.05) (Figure 3A). To confirm our results, real-time PCR was used to determine mRNA expression of TNF-α, IL-6, and IL-1β. The mRNA expression of these cytokines was decreased by PSS pretreatment compared with that in the cerulein group, especially in the higher dose group (*p* < 0.05) (Figure 3B). Western blot analysis was performed to determine the protein expression of these cytokines, which was visibly increased in the cerulein group (Figure 3C), consistent with the mRNA expression. However, the protein expression of these cytokines decreased in both PSS pretreatment groups, and PSS administered at 50 mg/kg was more effective, indicating that the effects of PSS on cerulein-induced AP were dose-dependent. The expression of inflammatory cytokines was determined by immunohistochemical staining (Figure 3D). The MPO results were consistent with the above results (*p* < 0.05) (Figure 3E). These findings provided strong evidence that pretreatment with PSS decreased the release of inflammatory cytokines.

### 2.4. PSS Pretreatment Decreased Apoptosis and Autophagy in Cerulein-Induced Acute Pancreatitis

**Figure 4:**
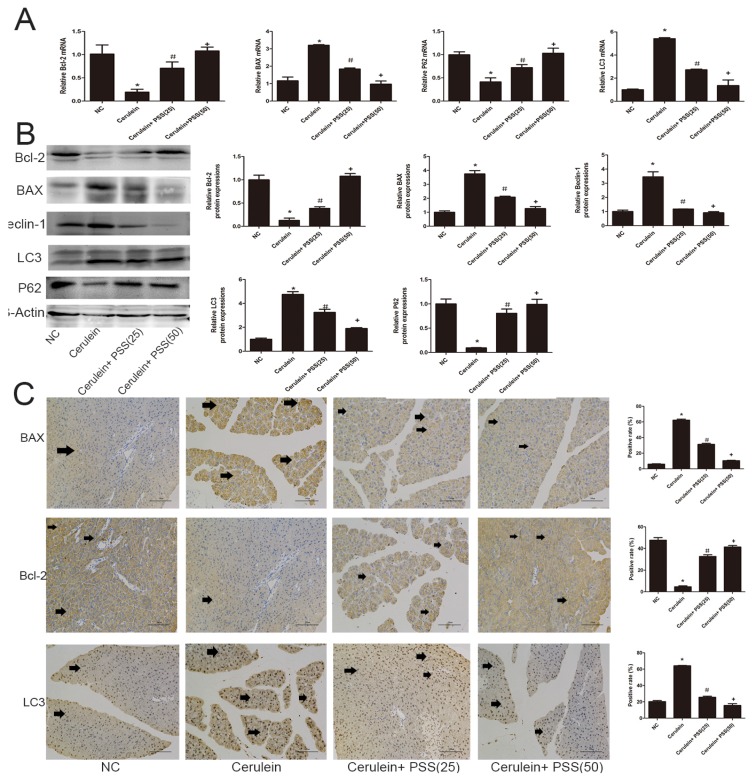

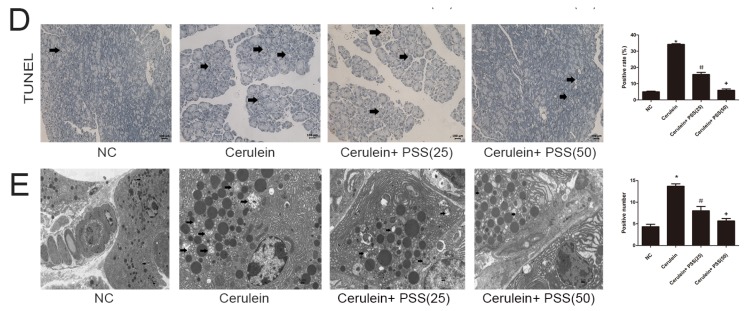
Effects of PSS on apoptosis and autophagy. (**A**) cDNA levels of Bcl-2, BAX, LC3, and p62 were measured by real-time PCR (*n* = 5, * *p* < 0.05 for NC versus cerulein, ^#^ *p* < 0.05 for cerulein + PSS (25 mg/kg) versus cerulein, and ^+^ *p* < 0.05 for cerulein + PSS (50 mg/kg) versus cerulein); (**B**) Protein expression of Bcl-2, BAX, LC3, Beclin-1 and p62 were determined by Western blotting and the gray values were calculated (*n* = 5, * *p* < 0.05 for NC versus cerulein, ^#^ *p* < 0.05 for cerulein + PSS (25 mg/kg) versus cerulein, and ^+^ *p* < 0.05 for cerulein + PSS (50 mg/kg) versus cerulein); (**C**) Immunohistochemistry staining (×200) showed the expression of BAX, Bcl-2, and LC3 protein. The ratio of brown area to total area was analyzed (*n* = 5, * *p* < 0.05 for NC versus cerulein, ^#^ *p* < 0.05 for cerulein + PSS (25 mg/kg) versus cerulein, and ^+^ *p* < 0.05 for cerulein + PSS (50 mg/kg) versus cerulein). The representative positive cells were indicated with arrows. Six fields were selected in each slice randomly, and were calculated by average positive rate; (**D**) TUNEL staining showed apoptotic cells in the four groups (×200). The percentage of TUNEL positive cells are analyzed (*n* = 5, * *p* < 0.05 for NC versus cerulein, ^#^ *p* < 0.05 for cerulein + PSS (25 mg/kg) versus cerulein, and ^+^ *p* < 0.05 for cerulein + PSS (50 mg/kg) versus cerulein). The representative apoptotic cells were indicated with arrows. Six fields were selected in each slice randomly, and were calculated by average positive rate; (**E**) Autophagosome formation was detected in pancreatic tissue by TEM (original magnification, ×10,000). The representative autophagosomes were indicated with arrows. Six fields were selected in each slice randomly, and were calculated by average positive rate.

We investigated the expression of Bcl-2, Bax, LC3, Beclin-1, and p62. Bcl-2 and Bax are markers of apoptosis while Beclin-1, LC3, and p62 play important roles in the process of autophagy. Real-time PCR and Western blot were used to determine the expression of apoptosis and autophagy markers at the mRNA and protein levels (Figure 4A,B). Bax, the pro-apoptotic protein, was upregulated in the AP group and downregulated in the PSS pretreatment group (*p* < 0.05). Bcl-2, an anti-apoptotic marker, was contrary to Bax results (*p* < 0.05). The TdT-mediated dUTP Nick-End Labeling (TUNEL) assay also showed clear pickup of apoptosis in PSS pretreated mice compared with cerulein-treated mice (*p* < 0.05) (Figure 4D). The expression of Beclin-1 and LC3 was assessed using RT-PCR and Western blotting, showing a statistically significant increase in the cerulein-treated group, while p62 showed the opposite pattern (*p* < 0.05). The formation of autophagosomes is a pivotal process in autophagy. Hence, electron microscopy was used to observe the ultrastructure of pancreatic cells. The number of phagophore, autophagosome and autolysosome were increased in the cerulein-treated group and decreased in the PSS pretreatment group (Figure 4E). Immunohistochemical changes confirmed these results regarding both apoptosis and autophagy (Figure 4C). These results provided strong evidence that PSS can attenuate pancreatic cell apoptosis and autophagy and protect the pancreas from pathological damage in cerulein-induced AP.

### 2.5. PSS Pretreatment Regulates the MEK/ERK Signaling Pathway in Cerulein-Induced Acute Pancreatitis

**Figure 5:**
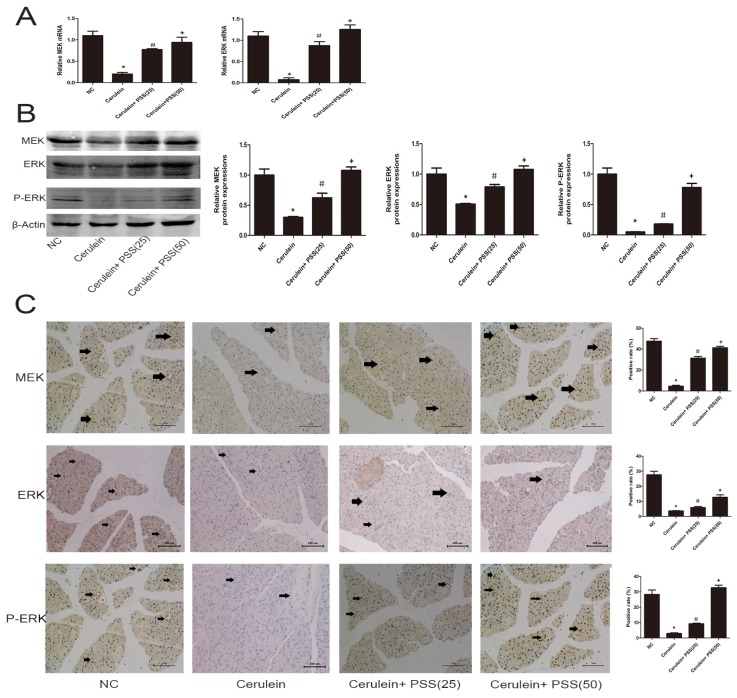
Effects of PSS on regulation of the MEK/ERK pathway. (**A**) The mRNA levels of MEK and ERK were determined by real-time PCR (*n* = 5, * *p* < 0.05 for NC versus cerulein, ^#^ *p* < 0.05 for cerulein + PSS (25 mg/kg) versus cerulein, and ^+^ *p* < 0.05 for cerulein + PSS (50 mg/kg) versus cerulein); (**B**) Protein expression of MEK, ERK and p-ERK were determined by Western blotting and the gray values were calculated (*n* = 5, * *p* < 0.05 for NC versus cerulein, ^#^ *p* < 0.05 for cerulein + PSS (25 mg/kg) versus cerulein, and ^+^ *p* < 0.05 for cerulein + PSS (50 mg/kg) versus cerulein); (**C**) Immunohistochemistry was used to detect MEK, ERK and p-ERK (original magnification, ×200). The ratio of brown area to total area was analyzed (*n* = 5, * *p* < 0.05 for NC versus cerulein, ^#^ *p* < 0.05 for cerulein + PSS (25 mg/kg) versus cerulein, and ^+^ *p* < 0.05 for cerulein + PSS (50 mg/kg) versus cerulein).

We showed that PSS can attenuate AP induced by cerulein through the inhibition of the release of pro-inflammatory cytokines, apoptosis and autophagy. However, the underlying mechanism remains unclear. Furthermore, the MEK/ERK signaling pathway has been confirmed to be associated with apoptosis and autophagy [8,9,10]. Therefore, we examined whether PSS could protect pancreatic tissues by regulating the MEK/ERK pathway. RT-PCR and Western blot analysis were used to determine the mRNA and protein levels of total MEK, ERK and the phosphorylation of ERK (Figure 5A,B), which were significantly decreased in the cerulein-treated group and clearly increased in the PSS-pretreated group (*p* < 0.05). As shown in Figure 5C, immunohistochemical staining was performed to detect the expression of MEK, ERK, and p-ERK. The results of RT-PCR, Western blotting and immunohistochemical staining suggested that PSS treatment can ameliorate pancreatic injury in cerulein-induced AP, in part through the MEK/ERK pathway.

## 3. Discussion

PSS, a drug used in cardiovascular diseases, has been commonly used in China for nearly 30 years. Previous studies have focused on its protective effects against thrombosis, but the anti-inflammatory potential of PSS remains undetermined. In this study, we found that PSS treatment caused a dose-dependent decrease in cytokines, autophagy and apoptosis. We also identified the molecular mechanism, and showed that PSS ameliorated the MEK/ERK pathway.

Pro-inflammatory cytokines such as TNF-α, IL-1β and IL-6 play a pivotal role in the initiation of AP. Activation of inflammatory cells is initiated during AP, exhibiting direct cytotoxicity or releasing proinflammatory cytokines, which mediate a systemic inflammatory response. A previous study has demonstrated that TNF-α plays an important role in the early stages of inflammation [11] and IL-6 is another vital mediator during the development of AP [12,13,14]. In the present study, we investigated the effect of PSS in a well-characterized model of AP induced by cerulein in mice. As shown in Figure 3, the protein and RNA levels expression of TNF-α, IL-6 and IL-1β increased sharply after cerulein injection, which was in accordance with previous studies. However, PSS reversed this effect, in a dose-dependent manner. Our results showed that PSS clearly ameliorates inflammation by inhibiting the production and release of inflammatory factors such as TNF-α, IL-6 and IL-1β. Apoptosis and autophagy are two important cellular processes with complex and intersecting protein networks. Cell apoptosis can be divided into two basic approaches: the exogenous approach and the endogenous approach. The induction of autophagy results in the formation of autophagosomes, which can produce several markers of autophagy, including LC3, Beclin-1, and p62. In the present study, the representative indicators of apoptosis and autophagy such as Bcl-2, Bax, Beclin-1, LC3 and P62 were detected by PCR, Western blot, immunohistochemistry, TUNEL staining and transmission electron microscopy. The expression levels of Bcl-2, P62 were decreased sharply in the AP group revealing that the effects of anti-apoptotic and anti-autophagy were attenuated. There was a significant increase in Bax, Beclin-1 and LC3, suggesting an increasing apoptosis and autophagy in the AP group as well. Nevertheless, pretreatment with PSS could reverse this effect. The Bcl-2 family can be divided into two types: one of which consists of anti-apoptotic proteins, including Bcl-2, Bcl-xL, Boo, CED-9, Bcl-w, Mcl-1, A1; the other consists of pro-apoptotic proteins, including Bax, Bak, Bad, Bcl-xs, Bid, Bok and Egl-1 [15,16,17]. The Bcl-2/Bax ratio is a decisive factor in cellular fate to a certain extent [18]. In the present study, pretreatment with PSS up-regulated the expression level of anti-apoptotic Bcl-2 and reduced the expression of Bax, indicating that PSS ameliorates inflammation by increasing the ability of cells to resist apoptosis and autophagy.

We investigated the pathways that regulate autophagy and apoptosis to further confirm which pathways were downstream of PSS. As shown in Figure 5, MEK, ERK and the phosphorylation of ERK in the AP group were markedly inhibited, which suggested that the MEK/ERK pathway was involved in cerulein-induced AP. After receptor ligation, Shc, Src homology (SH)-2, becomes associated with the C-terminus of the receptor. Shc recruits the GTP-exchange complex Grb2/Sos resulting in the loading of membrane-bound Ras with GTP. Ras-GTP phosphorylates Raf, which is responsible for MEK [19]. MEK phosphorylates ERK 1 and 2 [20]. ERK can translocate to the nucleus and phosphorylate additional transcription factors such as Bcl-2, Bad, Bim, Mcl-1 and Caspase 9. ERK can also phosphorylate Bad on S112, contributing to its inactivation [21]. This event contributes to the formation of homodimers and an anti-apoptotic response. ERK also phosphorylates Mcl-1 and Bim which allows Bcl-2, Bcl-xL and Mcl-1 to bind Bax and prevent Bax activation and the formation of Bax: Bax homodimers. In the present study, PSS facilitates the activation of the MEK/ERK pathway, increasing the anti-apoptotic capacity. Thus, apoptosis is inhibited [22,23]. Recently, it was found that the MEK/ERK cascade can also phosphorylate Caspase 9 on residue T125, which contributes to the inactivation of this protein. Meanwhile, Bcl-2 functions by interacting with Beclin-1 [24,25] and reduces the pro-autophagic activity of Beclin-1 [26], which is thought to be the mechanism of the inhibition of autophagy by PSS.

Interestingly, some research indicates that the activation of MEK/ERK up-regulates the level of Beclin-1 causing cytodestructive autophagy [9,27] and also an increase in the number of autophagosomes [10]. Moreover, MEK can bypass ERK to promote autophagy [27]. However, in our study, PSS attenuated pancreatic cell autophagy and protected the pancreas from pathological damage. Therefore, we hypothesized that overexpression of Bcl-2 attenuates the formation of the kinase complex Beclin-1-class III phosphatidylinositol 3-kinase (PI3KC3) which is essential for the formation of autophagosomes [28,29].

**Figure 6:**
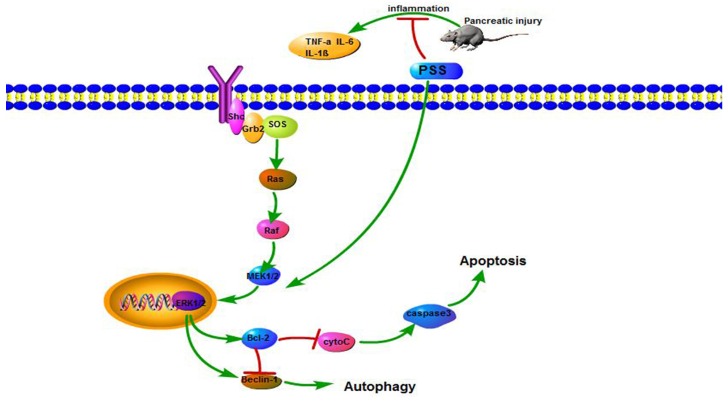
The mechanism of PSS action.

The present study determined the role of the MEK/ERK signal pathway by detecting the expression levels of gene MEK, ERK, p-ERK, Bcl-2, Bax and Beclin-1. The results showed a concentration-dependent increase in Bcl-2 and p-ERK. This indicated that the MEK/ERK signal pathway was significantly excited. Our findings provide new insight into the role of PSS in alleviating cerulein-induced AP by modulating the MEK/ERK pathway (Figure 6).

In AP induced by cerulein, PSS reduces apoptosis and autophagy by stimulating the MEK/ERK pathway. After receptor ligation, Shc, Src homology (SH)-2, becomes associated with the C-terminus of the receptor. Shc recruits the GTP-exchange complex Grb2/Sos resulting in the loading of membrane-bound Ras with GTP. Ras-GTP phosphorylates Raf, which is responsible for the MEK. MEK phosphorylates ERK 1 and 2. ERK can translocate to the nucleus and up-regulates the level of Beclin-1 and Bcl-2. Furthermore, Bcl-2 reduces the pro-autophagic activity of Beclin-1.

## 4. Materials and Methods

### 4.1. Reagents

PSS was purchased from Yabao Pharmaceutical (Yabao, Shanxi, China, catalogue numbers: 150103). Cerulein (catalogue numbers: c16350) and lipopolysaccharide (catalogue numbers: L of 9472) were purchased from Sigma-Aldrich (St. Louis, MO, USA). Antibodies were purchased from Cell Signaling Technology (Danvers, MA, USA) (antibodies against MEK(9126S), LC3(4108S), TNF-α(#3707S), IL-6(129126S) and IL-1β(12507S)), SAB (SAB, Shanghai, China) (antibodies against ERK(A1312), P-ERK(A1312)) and Proteintech (Proteintech, Chicago, IL, USA) (antibodies against p62(18420-1-AP), Beclin1 (11306-1-AP), Bcl-2 (12789-1-AP) and Bax (50599-2-Ig)), all of which were monoclonal antibodies from a rabbit anti-mouse source. The Amylase (F04772) and Lipase (F01220) ELISA kits were purchased from Shanghai Westang Bio-tech Co., Ltd. (Shanghai, China). TNF-α (MEC1003-2), IL-6 (MEC1008-2), and IL-1β (MEC1010-2) ELISA kits were purchased from Anogen-Yes Biotech Laboratories Ltd. (Anogen-Yes Biotech, Mississauga, ON, Canada). Myeloperoxidase (MPO) microplate test kits (A044) were purchased from Nanjing Jiancheng Bioengineering Institute (Jiancheng Biotech, Nanjing, China). The RNA polymerase chain reaction (PCR) kit (#AK4301) was purchased from Takara (Takara Biotechnology, Dalian, China).

### 4.2. Animals

Male Balb/C mice (18 ± 2 g, 6–8 weeks old) were purchased from Shanghai Laboratory Animal Co., Ltd. (SLAC, Shanghai, China). All animals were housed in a clean room at 23 ± 2 °C and humidity of 50% with a consistent light cycle (12 h:12 h). The mice were permitted free access to food and water. All animal experiments were approved by the National Institutes of Health Guidelines for the Care and Use of Laboratory Animals and were permitted by the Animal Care and Use Committee of Shanghai Tongji University, China.

### 4.3. Experimental Design

A total of 15 mice were randomly divided into three groups:

(1) normal control group (treated with saline solution)
(2) treated with 25 mg/kg PSS
(3) treated with 50 mg/kg PSS.

PSS was dissolved in saline solution. Five mice randomly selected from the three groups were killed. Serum and pancreatic tissues were obtained for analysis of cytokine levels, enzymes and pathological changes.

### 4.4. Drug Treatment

Acute pancreatitis was induced by ten intraperitoneal injections of cerulein (100 μg/kg). PSS was administered intraperitoneally (i.p.) (25 or 50 mg/kg) 1 h before the first cerulein injection in the PSS treatment group. The mice were randomly divided into four groups:

(1) Normal control (*n* = 12)
(2) Cerulein group (*n* =12): mice were injected with ten injections of cerulein (100 mg/kg, i.p. at intervals of 1 h) and 5 mg/kg lipopolysaccharide was injected intraperitoneally after the last injection.
(3) Low dose group (*n* = 12): mice were intraperitoneally injected with 25 mg/kg PSS 1 h before cerulein.
(4) High dose group (*n* =12): mice were intraperitoneally injected with 50 mg/kg PSS 1 h before cerulein.

Blood samples were taken when all animals were sacrificed at 12 h after the first injection of cerulein, a time point at which pancreatic damage had peaked based on our earlier findings [7]. Pancreatic tissue was rapidly removed and fixed in formalin for further studies.

### 4.5. Histological Analysis

A portion of the pancreas was fixed in 4% paraformaldehyde for 12 h, embedded in paraffin, and cut into 5-mm thick sections which were stained with hematoxylin and eosin, and the morphological changes were observed under a light microscope. The assessment of vacuolization, edema, acinar cell necrosis and inflammatory cell infiltration was carried out, as shown in Table 2.

### 4.6. Biochemical Analysis

Blood samples were obtained to determine serum amylase levels. Serum was obtained from the eyeballs of mice (centrifuged at 3000 g, 10 min, 4 °C). Serum amylase and lipase were measured using an enzyme-linked immunosorbent assay (ELISA) kit (Westang Bio-tech, Shanghai, China). Serum TNF-α, IL-1β and IL-6 were measured by ELISA using a commercial kit (Anogen-Yes Biotech, Canada).

### 4.7. Myeloperoxidase Activity

A portion of the pancreas was used for MPO activity assays. Myeloperoxidase is a neutrophil-specific enzyme, which is released into the extracellular space in the inflammatory state. Neutrophil sequestration in the pancreas was quantified by measuring tissue MPO activity according to the manufacturer’s protocol. MPO is a heme-containing enzyme that catalyzes the hydrogen peroxidase-mediated oxidation of halide ions to hypohalous acid (hypochlorous acid in this kit), which reacts with taurine to form taurine chloroamine, which reacts with the chromophore 5-thionitrobenzoic acid (TNB), resulting in the formation of the colorless product 5,5′-dithiobis-(2-nitrobenzoic acid (DTNB). The absorbance was then measured at 460 nm using a Beckman spectrophotometer (Beckman DU640B, Brea, CA, USA). Then, the MPO activity and the number of neutrophils were calculated. One unit of MPO activity is defined as the amount of enzyme that hydrolyzes the substrate and generates taurine chloramine to consume 1.0 millimoles of TNB per minute at 25 °C.

### 4.8. Real Time-PCR

mRNA transcripts in mouse pancreatic tissues were analyzed by real-time RT-PCR. Total RNA was isolated from the mouse pancreas using TRIZOL reagent (Invitrogen, Carlsbad, CA, USA) following the manufacturer’s protocol and was subjected to reverse transcription using the Prime Script RT reagent Kit (TaKaRa). SYBR Green quantitative RT-PCR was performed using a 7900HT Fast Real-time PCR system (ABI, Foster City, CA, USA) according to SYBR Premix EXTaq instructions (TaKaRa). Primer sequences for these biomarkers are shown in Table 3.

### 4.9. Immunohistochemistry

The prepared paraffin-embedded pancreas sections (5 μm) were heated at 60 °C for 1 h and then dewaxed and rehydrated using xylene and different concentrations of alcohol. Antigen retrieval was performed in citrate buffer for 10 min and then peroxidase quenched with 3% H_2_O_2_ in phosphate buffer saline (PBS) for 10 min. Sections were washed in PBS and blocked with 5% bovine serum album (BSA) for 30 min, then incubated with mouse antibodies at the following dilutions: TNF-α (1:100), IL-6 (1:100), Bax (1:100), Bcl-2 (1:100), LC3 (1:100), MEK (1:100), ERK (1:100) and P-ERK (1:100) (the specifications of antibody concentrations are 1 μg/μL (CST), 3 μg/μL (proteintech) and 1 μg/μL (SAB)) in a humid chamber overnight, washed with 0.1% Tween 20 in PBS three times (10 min each time) and incubated with biotinylated secondary antibody. The bound peroxidase was visualized following reaction for 3–15 min in a solution containing 3,3-diaminobenzidine (DAB), counterstained with hematoxylin, dehydrated and mounted. The slides were observed by light microscopy at a magnification of ×200. Data were analyzed using Image-Pro Plus software 6.0 (Media Cybernetics, Silver Spring, MD, USA).

### 4.10. Western Blot

Pancreatic tissues were lysed with RIPA lysis buffer supplemented with protease inhibitors (PI) and phenylmethanesulfonyl fluoride (PMSF). The protein concentration was determined using the bicinchoninic acid (BCA) protein assay (Kaiji, Shanghai, China). Equivalent amounts of total protein (80 μg) were boiled, separated using different concentrations of sodium dodecyl sulfate (SDS) polyacrylamide gels (SDS-PAGE) and then transferred to polyvinylidene difluoride (PVDF) membranes. Nonspecific binding was blocked with 5% nonfat milk for 1 h and incubated overnight at 4 °C with primary antibodies at the following dilutions: β-actin (1:1000), IL-1β (1:500), IL-6 (1:500), TNF-α (1:500), Bcl-2 (1:500), Bax (1:500), LC3 (1:500), p62 (1:500), MEK (1:500), ERK (1:500) and P-ERK (1:500). Membranes were washed with PBST three times (10 min each time) and then incubated with a secondary antibody (1:1000) for 1 h at room temperature. The membranes were then washed with PBST three times (10 min each time) and scanned using the Odyssey two-color infrared laser imaging system.

### 4.11. TUNEL Staining

Apoptosis of pancreatic tissues was assessed using the TUNEL assay. The prepared paraffin sections (5 μm) were dewaxed twice in xylene (5–10 min each time) and dehydrated with ethanol followed by digestion with 20 μg/mL proteinase K (Sigma-Aldrich) for 15 min at room temperature. The slides were washed four times, then incubated with 2% hydrogen peroxide in PBS for 5 min at room temperature. The slides were then washed twice, and then immersed in Terminal deoxynucleotidyl Transferase-containing buffer for 15 min. An antidigoxigenin antibody fragment carried a conjugated reporter enzyme (peroxidase) to the reaction sites, and then localized peroxidase generated an intense signal from the chromogenic substrate diaminobenzidine. The counterstain was methyl green. Apoptotic cells showing dark brown color were observed by optical microscopy.

### 4.12. Transmission Electron Microscopy

A portion of pancreatic tissue was placed in 2% glutaraldehyde buffer and postfixed in osmium tetroxide (OsO4). Then, it was viewed by electron microscopy (JEM 1230, JEOL, Tokyo, Japan) and the images were printed onto photographic paper.

### 4.13. Statistical Analysis

All results are expressed as means ± SD. Statistical analysis was performed with GraphPad Prism Software version 6.0 for Windows (GraphPad, San Diego, CA, USA). Quantitative data were compared using the Student’s *t* test (serum TNF-α, IL-6, and IL-1β, mRNA levels and protein expression of TNF-α, IL-6, IL-1β, Bcl-2, BAX, LC3, p62, MEK and ERK) and one-way analysis of variance (serum amylase, serum lipase, cytokines). Scoring data were analyzed by a non-parametric test (Kruskal–Wallis test) (pathological scores). *p* < 0.05 was considered statistically significant. All data had been tested and were consistent with the requirement of the Student’s *t* test, one-way analysis of variance and non-parametric test.

## 5. Conclusions

Our results demonstrated that the MEK/ERK signal pathway mediated cell apoptosis and autophagy in cerulein-induced AP. Then, we confirmed that PSS suppressed pancreatic injury caused by cerulein by inhibiting inflammatory factors such as TNF-α, IL-6, and IL-1β. Finally, PSS upregulated the MEK/ERK signal pathway, increasing the anti-apoptotic effect of Bcl-2 which blocked the pro-autophagic effect of Beclin-1. Overall, these findings suggest that PSS may be a promising potential therapeutic agent for AP.

## Figures and Tables

**Table 1:** Effect of PSS on pancreas pathological scores.

Pathologic Changes	Acinar Edema	Vacuolization	Inflammation	Acinar Cell Necrosis
Normal control	0.3 ± 0.1	0 ± 0	0.1 ± 0	0.1 ± 0.1
Cerulein	3.2 ± 0.5 *	0.4 ± 0.2 *	0.7 ± 0.2 *	0.8 ± 0.3 *
Cerulein+ astaxanthin (20)	1.6 ± 0.3 *^,^**	0.2 ± 0.1 *^,^**	0.3 ± 0.1 *^,^**	0.4 ± 0.2 *^,^**
Cerulein+ astaxanthin (40)	1.0 ± 0.3 *^,^**	0.1 ± 0.1 *^,^**	0.2 ± 0.1 *^,^**	0.2 ± 0.1 *^,^**

Eight fields of vision were randomly selected in each slice, and the average pathological scores were calculated as shown in Table 1. Data are expressed as the mean ± SD (*n* = 5 for each group). * *p* < 0.05 for Cerulein, Cerulein+ astaxanthin (20) and Cerulein+ astaxanthin (40) vs. normal control group. ** *p* < 0.05 for Cerulein+ astaxanthin (20) and Cerulein+ astaxanthin (40) vs. cerulein group.

**Table 2:** The criteria of pancreatic histological score.

Pathological Factors	Histological Score	Pathologic Change
	0	none
cell necrosis	1	<10% necrosis
	2	<40% necrosis
	3	>40%necrosis
	0	none
vacuolization	1	<20% acini with vacuoles
	2	<50% acini
	3	>50% acini
	0	none
inflammation	1	inflammatory cells present at interlobular areas
	2	present at intralobular areas
	3	present at interacini
	0	none
acinar edema	1	interlobular edema
	2	intralobular edema
	3	interacinar edema

**Table 3:** Primer sequences used for polymerase chain reactions.

Gene	Primer Sequence (5′-3′)
IL-1β	Forward: CGATCGCGCAGGGGCTGGGCGG
	Reverse: AGGAACTGACGGTACTGATGGA
IL-6	Forward: CTGCAAGAGACTTCCATCCAG
	Reverse: AGTGGTATAGACAGGTCTGTTGG
TNF-α	Forward: CAGGCGGTGCCTATGTCTC
	Reverse: CGATCACCCCGAAGTTCAGTAG
Bcl-2	Forward: GCTACCGTCGTCGTGACTTCGC
	Reverse: CCCCACCGAACTCAAAGAAGG
Bax	Forward: AGACAGGGGCCTTTTTGCTAC
	Reverse: AATTCGCCGGAGACACTCG
LC3	Forward: GACCGCTGTAAGGAGGTGC
	Reverse: AGAAGCCGAAGGTTTCTTGGG
P62	Forward: GAGGCACCCCGAAACATGG
	Reverse: ACTTATAGCGAGTTCCCACCA
MEK	Forward: TCCTCACCAGGTTTAGAATTGC
	Reverse: GCGAGTTTCTCACGTCGGA
ERK	Forward: ACTGCTGGGCATAACGCTTTT
	Reverse: GAGGAGGATCTTGAGAGCCTT
β-actin	Forward: GGCTGTATTCCCCTCCATCG
	Reverse: CCAGTTGGTAACAATGCCATGT
